# Is red distribution width a valid tool to predict impaired iron transport in heart failure?

**DOI:** 10.3389/fcvm.2023.1133233

**Published:** 2023-04-11

**Authors:** Jeness Campodonico, Ermes Carulli, Francesco Doni, Gerardo Lo Russo, Daniele Junod, Margherita Gaudenzi Asinelli, Alice Bonomi, Fabiana De Martino, Carlo Vignati, Beatrice Pezzuto, Piergiuseppe Agostoni

**Affiliations:** ^1^ Centro Cardiologico Monzino, IRCCS, Milano, Italy; ^2^ Department of Clinical Science and Community Health, Cardiovascular Section, University of Milan, Milan, Italy

**Keywords:** iron metabolism, heart failure, red cell distribution width (RDW), iron deficiency, iron metablism disorder

## Abstract

**Background:**

Impaired iron transport (IIT) is a form of iron deficiency (ID) defined as transferrin saturation (TSAT) < 20% irrespective of serum ferritin levels. It is frequently observed in heart failure (HF) where it negatively affects prognosis irrespective of anaemia.

**Objectives:**

In this retrospective study we searched for a surrogate biomarker of IIT.

**Methods:**

We tested the predictive power of red distribution width (RDW), mean corpuscular volume (MCV) and mean corpuscular haemoglobin concentration (MCHC) to detect IIT in 797 non-anaemic HF patients.

**Results:**

At ROC analysis, RDW provided the best AUC (0.6928). An RDW cut-off value of 14.2% identified patients with IIT, with positive and negative predictive values of 48 and 80%, respectively. Comparison between the true and false negative groups showed that estimated glomerular filtration rate (eGFR) was significantly higher (*p* = 0.0092) in the true negative vs. false negative group. Therefore, we divided the study population according to eGFR value: 109 patients with eGFR ≥ 90 ml/min/1.73 m^2^, 318 patients with eGFR 60–89 ml/min/1.73 m^2^, 308 patients with eGFR 30–59 ml/min/1.73 m^2^ and 62 patients with eGFR < 30 ml/min/1.73 m^2^. In the first group, positive and negative predictive values were 48 and 81% respectively, 51 and 85% in the second group, 48 and 73% in the third group and 43 and 67% in the fourth group.

**Conclusion:**

RDW may be seen as a reliable marker to exclude IIT in non-anaemic HF patients with eGFR ≥60 ml/min/1.73 m^2^.

## 1. Introduction

Iron Deficiency (ID) is frequently reported affecting more than 2 billion people worldwide (1). It is the leading cause of anaemia, accounting for up to 50% of cases in women, and it has been reported as a comorbidity in 60% of systolic heart failure (HF) patients (2). In HF, ID is associated with a worse prognosis by fostering circulatory failure, impairing functional capacity, and contributing to frailty (3–5). It can be responsible for fatigue, impaired exercise performance, and a reduction of work productivity in adults, while in children it could hamper cognitive development (3, 4). In fact, iron is implicated in the energy production of cells *via* Krebs cycle and the mitochondrial respiratory chain, and it contributes to DNA synthesis and neurotransmitter production. These are the reasons why an impaired iron metabolism can lead to systemic consequences (6).

As reported in several guidelines (7, 8) the diagnosis of ID is based on the assessment of serum ferritin and transferrin saturation (TSAT) and specifically ID definition requires serum ferritin <100 µg/L or ferritin between 100 and 299 µg/L and TSAT <20%. However, a specific form of ID, namely impaired iron transport (IIT), has been proven to worsen prognosis in HF the most. ITT definition requires TSAT < 20% irrespective of serum ferritin levels (9, 10).

Current European Society of Cardiology (ESC) Guidelines recommend that HF patients be periodically screened for ID (7); nonetheless, this is not regularly done in the routine clinical practice, as highlighted by registry studies (11), mainly due to practical reasons and the economic burden associated to the need for repeated screening of all HF patients. Therefore, the aim of this study is to identify a reliable, frequently available, easy to obtain and cheap marker of IIT and consequently to select HF patients most deserving further investigation of their iron status.

## 2. Material and methods

We retrospectively collected data of 1,496 patients hospitalized for HF between January 2016 and November 2021 in the HF Unit of Centro Cardiologico Monzino (Milan, Italy).

The study inclusion criteria were: HF hospitalization, presence of ID screening and absence of anaemia. Patients were defined as anaemic when haemoglobin levels were below 13 g/dl in males and 12 g/dl in females according to WHO (World Health Organization) definition of anaemia (12). Study exclusion criteria were patients aged less than 18, pregnant or breast-feeding women, recent blood trasfusion, acute and chronic inflammatory conditions as well as patients hospitalized for cardiogenic shock or HF linked to acute myocardial infarction or myocardities.

A total of 797 patients fulfilled the study inclusion/exclusion criteria and their data were considered in the present analysis. As part of the routine laboratory analysis, when patients were in stable clinical conditions, we measured haemoglobin, haematocrit, red blood cell indices (red cell distribution width, RDW; mean corpuscular volume, MCV; and mean corpuscolar haemoglobin concentration, MCHC), iron status (serum iron; ferritin; transferrin saturation, TSAT and total iron binding capacity, TIBC) and renal function [creatinine and estimated glomerular filtration rate, eGFR, by MDRD formula (13)].

At the time of hospital admission, all patients underwent complete cardiac ultrasound evaluation including left ventricular ejection fraction (LVEF) by biplane method (14).

The study was approved by the local ethics committee (CCM04_21 PA).

### 2.1. Statistical analysis

Continuous variables are presented as mean and standard deviation (SD) if normally distributed, otherwise as median and interquartile range (IQR). Categorical variables are reported as frequencies and percentages. Continuous variables between groups were compared by *t*-test or Mann-Whitney *U*-test, while categorical variables were compared by Chi-square test or Fisher's exact test, as appropriate.

Through estimation of the area under the curve (AUC) of the ROC curve, the predictive ability of RDW, MCV and MCHC to detect patients with possible IIT was assessed. These parameters were arbitrarily chosen because part of the routine hematopoietic laboratory analysis and all were presumed to be linked to iron status (15).

After finding the best parameter, it was investigated whether any further predictive ability was reached by a combination of the above reported variables.

A cross-validation procedure was employed to calculate the best cut-off of selected parameter(s) to identify IIT patients. The study population was randomly split in half 200 times, the best cut-off by Euclidean distance was estimated in the first arm (training set), and its sensitivity and specificity were subsequently tested in the second half (testing set). The mean value of each cut-off was considered.

Thereafter we compared the true negative and false negative groups to identify the demographic (age and sex) and laboratory (creatinine, eGFR, MCV and MCHC) features.

Since we found out that RDW's relationship with TSAT levels significantly changed with the eGFR value, a further analysis was conducted by dividing the study population according to eGFR: <30 ml/min/1.73 m^2^, 30–60 ml/min/1.73 m^2^, 60–90 ml/min/1.73 m^2^ and ≥90 ml/min/1.73 m^2^. In each group sensitivity, specificity, positive predictive value and negative predictive value were calculated.

A *p*-value < 0.05 was considered statistically significant. Statistical analyses were performed with SAS software, version 9.4 (SAS Institute, Cary, NC, USA).

## 3. Results

Seven-hundred-ninety-seven non-anaemic HF patients were identified and evaluated in the present report. Two-hundred-sixty-four cases, 33.1% of the study population, had TSAT values < 20% and therefore were classified as IIT patients.

Demographic and clinical characteristics of the entire population and grouping patients according to the presence/absence of IIT are displayed in Table 1.

**Table 1:** General carachteristics of the population and differences between patients with impaired iron transport (IIT) vs. patients without.

	Total population (*n* = 797)	Impaired iron transport (IIT)		
		Absent (*n* = 535)	Present (*n* = 262)	*p*
Age (year)	68 ± 14	68 ± 14	68 ± 14	0.9064
Weight (kg)	76 ± 17	77 ± 16	75 ± 17	0.1218
Height (m)	1.69 ± 0.09	1.69 ± 0.09	1.67 ± 0.10	0.0171
BMI (kg/m^2^)	26 ± 5	27 ± 5	26 ± 5	0.6216
Hb (g/dl)	14.1 (13.3–15.2)	14.4 (13.4–15.4)	13.7 (13.1–14.6)	<.0001
Hct (%)	42.8 ± 4.2	43.0 ± 4.0	42.4 ± 4.5	0.108
RDW (%)	14.0 (13.2–15.3)	13.7 (13.0–14.7)	14.8 (13.8-16.1)	<.0001
MCV (fl)	89.6 (86.1–92.7)	90.2 (87.0–93.2)	88.0 (84.5-91.3)	<.0001
MCHC (%)	33.5 ± 1.5	33.7 ± 1.4	32.0 ± 1.4	<.0001
Serum iron (µg/dl)	76 (57–99)	92 (77–114)	51 (40–63)	<.0001
Serum ferritin (µg/L)	102 (55–186)	119 (71–214)	67 (32–130)	<.0001
Transferrin (mg/dl)	264 ± 52	253 ± 43	283 ± 60	<.0001
TSAT (%)	24 (16–31)	28 (24–35)	15 (12–17)	<.0001
TIBC (µg/dl)	323 (290–369)	314 (283–346)	354 (309–406)	<.0001
Creatinine (mg/dl)	1.10 (0.91–1.38)	1.07 (0.90–1.32)	1.18 (0.95–1.47)	0.0013
eGFR (MDRD) (ml/min/1.73 m^2^)	64 ± 23	66 ± 24	59 ± 22	<.0001
Lvef (%)	42 ± 15	42 ± 15	41 ± 16	0.2815

Data are reported as mean ± standard deviation or median (interquartile range). BMI, body mass index; eGFR, estimated glomerular filtration rate; Hb, haemoglobin; Hct, haematocrit; LVEF, left ventricular ejection fraction; MCHC, mean corpuscular haemoglobin concentration; MCV, mean corpuscular volume; MDRD, modification of diet in renal disease; RDW, red distribution width; TIBC, total iron binding capacity; TSAT, transferrin saturation.

**Figure 1:**
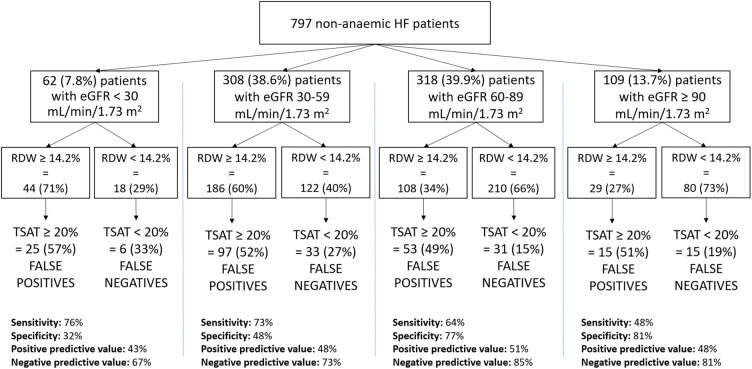
(**1**) ROC analysis of RDW, MCHC and MCV for the detection of IIT. IIT: impaired iron transport; MCHC, mean corpuscular haemoglobin concentration; MCV, mean corpuscular volume; RDW, red cell distribution width; ROC, receiver operating characteristic. (**2**) ROC analysis of RDW, RDW + MCHC and RDW + MCV for the detection of IIT. IIT, impaired iron transport; MCHC, mean corpuscular haemoglobin concentration; MCV, mean corpuscular volume; RDW, red cell distribution width; ROC, receiver operating characteristic.

We tested RDW, MCV and MCHC as possible IIT predictors: RDW performed the best with an AUC of 0.6891 (Figure 1(1)). Of note, any combination of RDW with MCV and MCHC did not allow any further significant improvement in the RDW IIT predictive power (Figure 1). In the overall population of non-anaemic HF patients, an RDW ≥ 14.2% resulted as the best cut-off to identify patients with IIT with a sensitivity of 68% and a specificity of 64%; positive predictive value and negative predictive value are 48% and 80%, respectively.

In order to find other parameters which could contribute to improve the test's sensitivity, we performed a subgroup analysis on the true negative (345 subjects) and false negative (85 subjects) patients. Among the parameters analysed, eGFR by MDRD formula was significantly higher (*p *= 0.0092) in the true negative (72 ± 22 ml/min/1.73 m^2^) vs. false negative group (65 ± 23 ml/min/1.73 m^2^). Borderline significant differences in the two groups resulted also for gender (*p* = 0.0495) (Table 2).

**Table 2:** Subgroup analysis of the true negative and false negative populations.

	True negatives (*n* = 345)	False negatives (*n* = 85)	*p*
Male sex (%)	245 (71%)	51 (60%)	0.0495
Age (year)	65 ± 14	65 ± 15	0.9554
MCV (fl)	90.2 (87.6–93.2)	89.9 (86.8–92.1)	0.1317
MCHC (g/dl)	33.9 (33.6–34.5)	33.6 (33.2–34.4)	0.2681
Creatinine (mg/dl)	1.01 (0.86–1.2)	1.07 (0.87–1.32)	0.0945
eGFR MDRD (ml/min/1.73 m²)	72 ± 22	65 ± 23	0.0092

Data are reported as the number of cases and percentage, as mean ± standard deviation or median (interquartile range). eGFR, estimated glomerular filtration rate; IQR, interquartile range; MCHC, mean corpuscular haemoglobin concentration; MCV, mean corpuscular volume; RDW, red distribution width.

**Figure 2:**
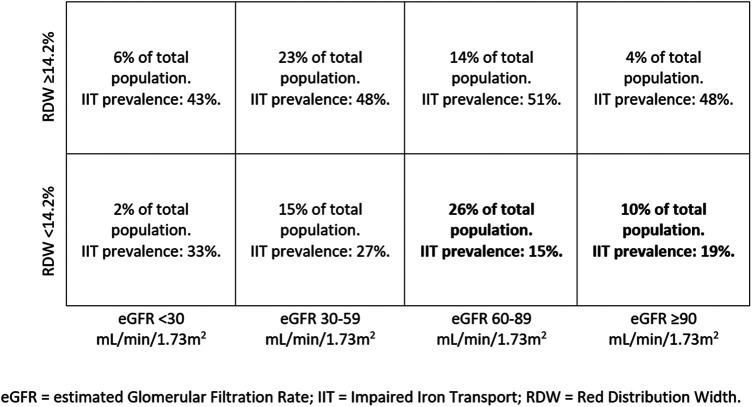
Sensitivity, specificity, positive predictive value and negative predictive value in the study population, divided based on the eGFR (MDRD) values. HF, heart failure; eGFR, estimated glomerular filtration rate; RDW, red cell distribution width; TAST, transferrin saturation.

Thereafter we grouped our patients according to eGFR. Sixty-two (7.8%) patients had eGFR <30 ml/min/1.73 m^2^, 308 (38.6%) patients had eGFR ranging between 30 and 60 ml/min/1.73 m^2^, 318 (39.9%) patients had eGFR between 60 and 90 ml/min/1.73 m^2^ and 109 (13.7%) patients had eGFR ≥90 ml/min/1.73 m^2^. On average, RDW decreases as renal function improves. Specifically, RDW levels were 15.8 ± 2.7% in the group with eGFR <30 ml/min/1.73 m^2^, 14.6 ± 3.8% with eGFR between 30 and 59 ml/min/1.73 m^2^, 14.3 ± 4.9% with eGFR between 60 and 89 ml/min/1.73 m^2^ and 13.8 ± 1.3% with eGFR ≥90 ml/min/1.73 m^2^. IIT was identified in the eGFR group <30 ml/min/1.73 m^2^ with a sensitivity of 76%, specificity of 32%, PPV of 43% and NPV of 67%, in eGFR ≥30 and <60 ml/min/1.73 m^2^ group, they were 73%, 48%, 48% and 73% respectively, in the eGFR ≥60 and <90 ml/min/1.73 m^2^ group, they were 64%, 77%, 51% and 85% respectively, in the eGFR ≥90 ml/min/1.73 m^2^ group, they were 48%, 81%, 48% and 81% respectively (Figure 2). Accordingly, an RDW value ≥14.2% identified non-anaemic HF patients with a TSAT < 20% with a sensitivity increasing in parallel with renal function worsening.

## 4. Discussion

In this study we found that IIT is present in around 1/3 of non-anaemic HF patients and IIT presence can be assumed in case of elevated anisocytosis as suggested by a high RDW value. Specifically, a value of 14.2% is the most precise RDW cut-off to suggest IIT in non-anaemic HF patients. The highest specificity of RDW as an indicator of IIT was found in non-anaemic HF patients with preserved renal function with an eGFR ≥90 ml/min/1.73 m^2^. Similarly, a very high negative predictive value was observed in HF patients with normal or moderately reduced eGFR, meaning that in these patients the presence of ITT can be reasonably excluded in patients with normal RDW. On the other hand, the negative predictive value of the proposed cut-off moderately decreases with eGFR ranging between 60 and 30 ml/min/1.73 m^2^, and even more under 30 ml/min/1.73 m^2^.

The population we analyzed consists of unselected, consecutive HF patients hospitalized for HF worsening or need for HF workup. In this population, IIT is a relatively common finding, even in the absence of anaemia. Unfortunately, an evaluation of iron status in non-anaemic HF patients is recommended by guidelines but not frequently performed in clinical practice. Indeed, in the present population of non-anaemic hospitalized HF patients, IIT prevalence is around 33% (262 out of 797 cases). In addition, IIT is the form of ID which is most linked with a worse prognosis in HF patients, as previously shown by a few reports (9, 10). Moreover, cardiopulmonary exercise test studies have clearly demonstrated that IIT is associated with impaired functional capacity as shown by reduced peak VO_2_ and high VE/VCO_2_ slope, being both established tools for defining prognosis in HF patients (4, 16). Accordingly, early IIT identification is important to properly assess HF patient's prognosis, the need for tailored therapy and follow-up strategies. On top, precocious detection of IIT is helpful for promptly initiating iron supplementation, which has been proven beneficial in terms of reduction in recurrent cardiovascular hospitalizations in patients with systolic HF and ID (17). Non-anaemic HF patients with IIT were characterized by lower Hb levels, albeit within the normal range, reduced iron parameters and lower kidney function. These data suggest that in non-anaemic HF patients the tight link between iron metabolism, red blood cell characteristics and kidney function speaks in favor of a continuum between iron metabolism, kidney function and hematopoietic status in HF.

RDW is calculated as the standard deviation of MCV ÷ MCV × 100 and is the most common indicator of anisocytosis, i.e., a high variability of red blood cell diameter generally due to ineffective erythropoiesis. An elevated RDW value is frequently associated with chronic illness, as in HF, and associated to disease progression (18, 19). It is linked to iron, folate or vitamin B12 deficiency, red cell early destruction, and an inflammatory status which leads to bone marrow dysfunction, hepatic congestion and hematological disorders (20, 21). As regards chronic HF, RDW has been reported to be a strong tool for HF prognosis (21–24) alone or in combination with NT-proBNP (25). Of note RDW is usually part of the data provided when a complete red blood cell count is requested and therefore is frequently available without a specific request and further economic burden.

Our findings extend those of Van Craenenbroeck et al. who showed that RDW may be associated with ID in HF patients with a reduction of RDW after ID treatment with ferric carboxymaltose (20). Indeed, we showed for the first time that the NPV of an RDW cut-off of 14.2% for detecting IIT progressively reduces in parallel with eGFR reduction and that the incidence of high RDW increases as eGFR decreases. This means that, in subjects with normal or slightly reduced eGFR, a low RDW is associated with IIT in very few cases. Vice-versa, in patients with low eGFR RDW is unable to reliably predict IIT and needs to be directly assessed.

**Figure 3:**
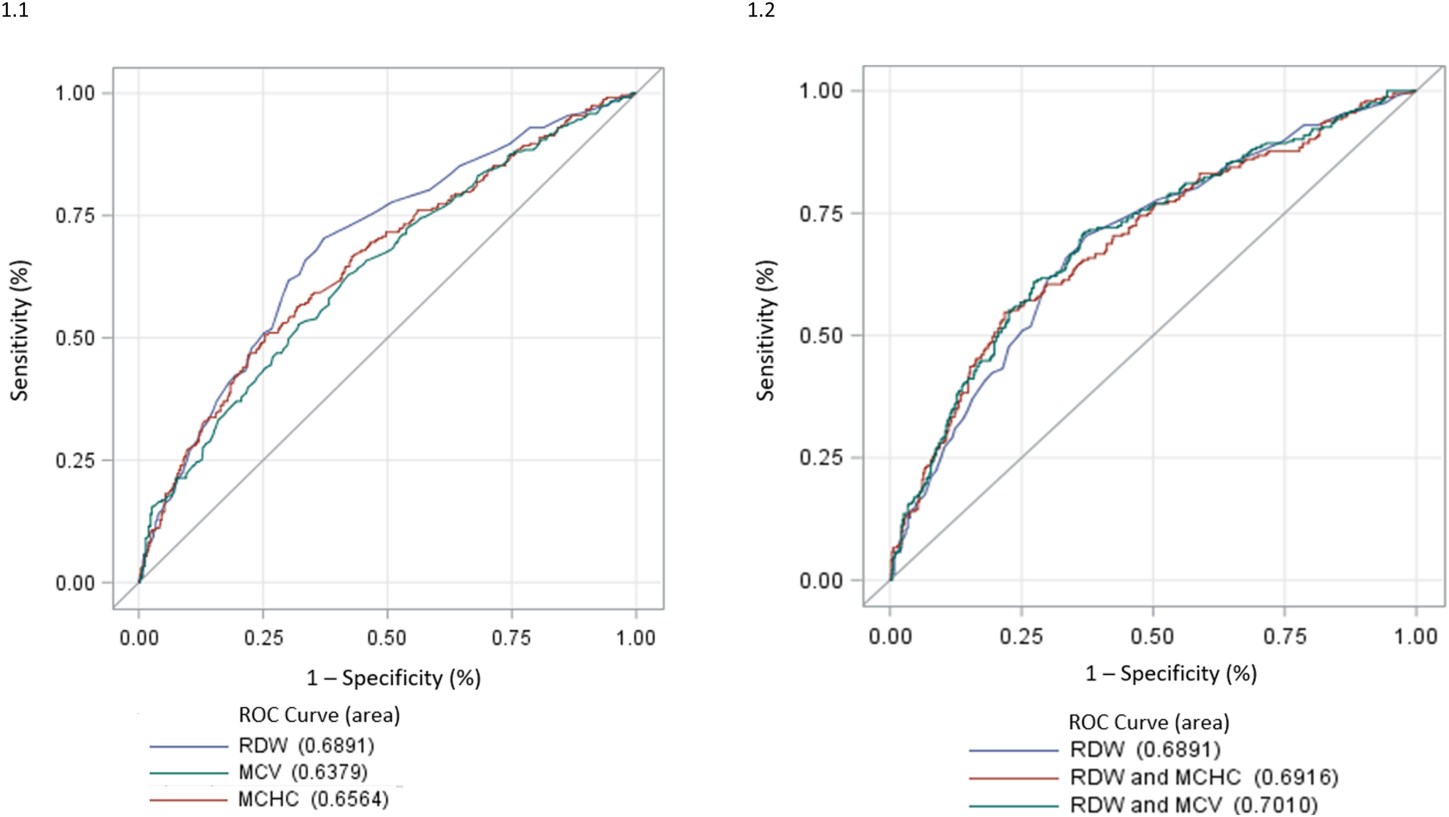
Percentage of patients with RDW </≥ 14.2% divided by eGFR values, with IIT prevalence in each group. An RDW cut-off of 14.2% performs best in excluding IIT in patients with eGFR ≥60 ml/min. eGFR, estimated glomerular filtration rate; IIT, impaired iron transport; RDW, red distribution width.

This study presents confirmatory and novel data about ID in HF and specifically as regards IIT, the form of ID which is related to the worst exercise performance and HF prognosis (9). Of note, differently from ID, data about IIT in non-anaemic HF patients are rarely reported and IIT prevalence still incompletely defined. We confirmed, in a sizable population of hospitalized non-anaemic HF patients, the high prevalence of IIT (about 1/3 of patients) and showed for the first time that an RDW value > 14.2% reliably predicts IIT. This finding has relevant clinical implication. Indeed, while ESC and other guidelines (7, 26) recommend ID evaluation in HF patients, it is not suggested how often this evaluation should be repeated. Moreover, albeit i.v. ferric carboxymaltose treatment has been reported to be effective in reducing recurrent hospitalizations in systolic HF patients with ID (17), the duration of this positive action is undefined. Therefore, the potential for repeated evaluations of IIT is clinically relevant. Of major importance is the observation that, in chronic HF patients with preserved or almost preserved renal function and RDW <14.2%, the presence of IIT is rare. However, it must be emphasized that we do not suggest avoiding ID evaluation and using RDW instead. We simply suggest that, when ID data are not available, RDW may be used to have an idea about ID presence or absence. Particularly, ID may be reliably excluded in case of low RDW and normal or moderately impaired renal function (Figure 2). The schema reported in Figure 3 may be useful for a practical point of view, showing the low risk of IIT in patients in the lower right quadrants representing 26% and 10% of the study population, respectively. In the other patient groups, IIT risk is higher. Accordingly, RDW measurement, which is frequently available and cheap, has a definite role in the follow up of HF patients as regards IIT, which is the most dangerous form of ID (9).

The present study has a few limitations which should be acknowledged. First, the retrospective nature of the study. Second, the population of non-anaemic HF patients we studied is relatively small and larger population studies are needed. Third, HF patients were recruited regardless of the ejection fraction value, the HF etiology or severity therefore we do not know whether our findings could apply differently in specific HF phenotypes such as, for instance, patients with low, middle range and preserved ejection fraction. Similarly, we do not know whether our findings could apply in anaemic HF patients or in HF patients with different comorbidities such as hepatic, renal, neurological diseases. Fourth, we analyzed IIT and not all forms of ID. Accordingly, we do not know whether RDW is associated with ID in specific settings such as in ID patients with TSAT > 20%. Fifth, we used cut-off values for grouping patients according to RDW and renal function. Albeit clinically useful, it must be underlined that cut-off values of any parameters are not reliable to evaluate measurements that are very close to the cut-off itself. Sixth, eGFR was obtained by MDRD formula. We recognized that other formulas such as CKD-EPI may be effectively used. However, MDRD is frequently used in HF. Furthermore, no patients have acute kidney injury, which is the condition where MDRD fails more frequently to properly predict eGFR. Finally, we have not evaluated whether an effective IIT treatment influences RDW, albeit some data show a reduction of RDW in ID patients after treatment (20).

In conclusion, albeit several HF guidelines recommend ID detection, this is not routinely performed in clinical practice. The present study suggests that RDW, a cheap and frequently available parameter, is a reliable predictor of IIT in HF patients. The presence of an elevated RDW value reinforces the need for ID evaluation in non-anaemic HF patients, while a normal RDW, particularly in subjects with preserved kidney function, is rarely associated with IIT being the latter the strongest predictive element of the present analysis.

## Data Availability

Raw data will be available upon request at zenodo.org and/or the corresponding authors.
